# EOG Signal Classification with Wavelet and Supervised Learning Algorithms KNN, SVM and DT

**DOI:** 10.3390/s23094553

**Published:** 2023-05-07

**Authors:** Sandy Nohemy Hernández Pérez, Francisco David Pérez Reynoso, Carlos Alberto González Gutiérrez, María De los Ángeles Cosío León, Rocío Ortega Palacios

**Affiliations:** 1 Master’s Degree in Information and Communications Technologies, Universidad Politécnica de Pachuca (UPP), Zempoala 43830, Mexico; sandyhdez@micorreo.upp.edu.mx; 2 Center for Research, Innovation and Technological Development UVM (CIIDETEC-UVM), Universidad del Valle de México, Querétaro 76230, Mexico; calberto.gonzalez@uvmnet.edu; 3 Direction of Research, Innovation and Graduate Studies, Universidad Politécnica de Pachuca (UPP), Zempoala 43830, Mexico

**Keywords:** EOG, wavelet transform, classifier algorithms

## Abstract

The work carried out in this paper consists of the classification of the physiological signal generated by eye movement called Electrooculography (EOG). The human eye performs simultaneous movements, when focusing on an object, generating a potential change in origin between the retinal epithelium and the cornea and modeling the eyeball as a dipole with a positive and negative hemisphere. Supervised learning algorithms were implemented to classify five eye movements; left, right, down, up and blink. Wavelet Transform was used to obtain information in the frequency domain characterizing the EOG signal with a bandwidth of 0.5 to 50 Hz; training results were obtained with the implementation of K-Nearest Neighbor (KNN) 69.4%, a Support Vector Machine (SVM) of 76.9% and Decision Tree (DT) 60.5%, checking the accuracy through the Jaccard index and other metrics such as the confusion matrix and ROC (Receiver Operating Characteristic) curve. As a result, the best classifier for this application was the SVM with Jaccard Index.

## 1. Introduction

The ocular muscles produce an electrical potential difference with an origin between the corneal pigment epithelium and the retina [1]. This differential is known in [2] as the Electrooculography (EOG) signal. EOG is obtained using silver electrodes placed superficially on the face, registering the horizontal channel (left-right movements) and the vertical channel (up and down movements).

The use of EOG in [3,4,5,6,7] the literature reviewed shows mostly its application by users with motor disabilities, turning their eyes to communicate. Therefore, the monitoring of biological signals such as EOG allows the integration of everyday objects, as mentioned in [8], where writing is performed by selecting a group of limited words for the response of short sentences; another application handled in [9] has is the electrical control of a wheelchair by eye movements; as well as in [10], the recognition of eye movement by different parameters detected in the signal, when visualizing different abstract images; the movement of the mouse cursor when receiving a signal from both eyes is described in [11], and the directional control of a robot in [12] were designed by a method based on saccadic movements and eye reflexes that were obtained as the average speed, maximum speed and voltage range in the developed model and did not include the fixed gaze and blinking movement.

EOG signal parameters are mostly detected at low frequencies, in a bandwidth of 0.5 to 50 Hz. The eye movement classification is based on algorithms that implement the calculation of the signal derivative; each algorithm targets different parameters (average velocity, maximum velocity, average acceleration, maximum acceleration, amplitude range, average velocity, maximum velocity, filtered average acceleration, filtered maximum acceleration, amplitude range, signal coefficients, four order of the polynomial, fit and slope of the signal) of the EOG signal amplitude for class classification; these parameters can be obtained in the time domain.

Many classification algorithms have been used to identify eye movements in EOG signals, including fixations and muscle denoising. Due to their relevant results presented, K-Nearest Neighbor (KNN), Support Vector Machine (SVM) and Decision Tree (DT) are reported as simpler and more efficient algorithms for EOG classification. In [13], the classification of an EOG signal using power spectral density (PSD) is described, these features are the neural network input and support vector machine (SVM) algorithm, and the performance of the combination of these methods achieves a classification accuracy of 69.75%.

There has been research on the combination of KNN and SVM algorithms; for example, [14] used image classification, relying on a spectral feature parameter input, to reduce the time of classification and data selection.

In [15], the authors classified 23 feature emotions such as anger, fear, happiness and sadness, obtaining an accuracy of 75.15% for KNN and 80% for SVM, comparing two algorithms to find the classifier with the highest result.

In the implementation of physiological signals in the time domain addressed in [16], the efficiency of the classification accuracy with the confusion matrix was tested. Comparing the results obtained from both classifiers, it was concluded that the SVM algorithm obtained 60% classification with the handling of six parameters.

In the case of [17], the extraction of 16 parameters of the EOG movement signal in the time domain was applied, taking 12 test subjects as a sample; the authors were classified with DT (95.4), KNN (99.6%) and SVM (99.1%) algorithms, and it was reported that by using the ROC curve, it was shown that the best result could be obtained by the KNN algorithm.

The following sections describe the EOG signal acquisition protocol, data preprocessing using Wavelet Transform [18,19], selecting of the Mother Wavelet using entropy and statistical parameters such as variance, peak magnitude ratio (RMS) and peak amplitude (AMP), the average and median frequency of the total samples, and the classification of five eye movements (left, right, up, down and blink) is described using the supervised learning algorithms KNN, DT and SVM, to validate the efficiency of each classifier metrics such as the ROC curve [20], confusion matrix [21] and Jaccard Index [22], which were implemented.

## 2. Materials and Methods

### 2.1. EOG Acquisition

**Figure 1:**
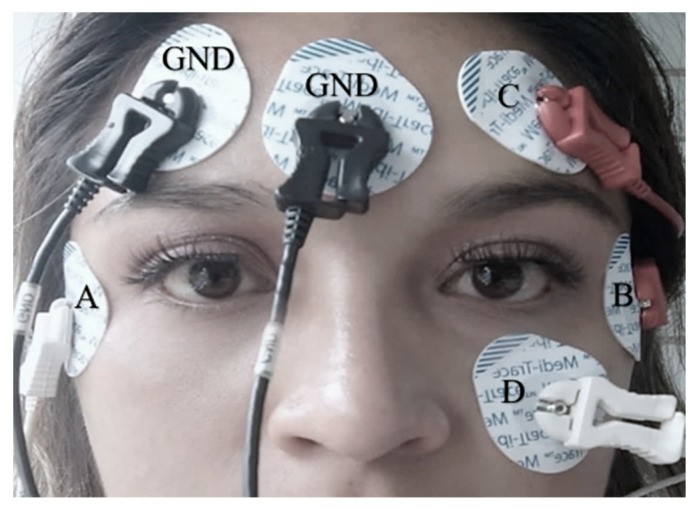
The electrodes were placed superficially on the participant’s face. The GND electrodes had the function of being the reference of the horizontal and vertical lead (placed on the forehead or earlobe). A and B are the electrodes of the horizontal channel, C and D are the electrodes of the vertical channel.

**Figure 2:**
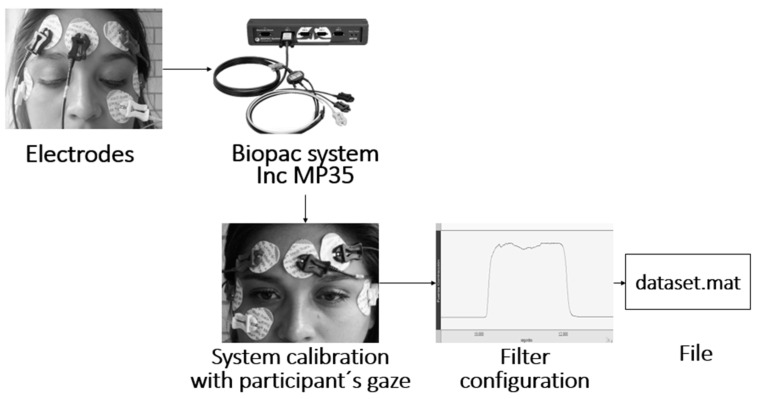
Signal recording process that was saved in a file with extension .mat that served as an input to the classification algorithms; the process started with the placement of electrodes next to the acquisition system, followed by calibration tests, obtaining the signal and ending with the storage of a data file.

The EOG signal was acquired using a recording protocol with 5 surface mount Ag/AgCl electrodes, as shown in Figure 1; the recording had a bandwidth between 0.5 and 50 Hz with a gain amplitude of 100, which expressed the ratio between the amplitude of an output signal with respect to the input signal, and a resolution of 11 bits using the Biopac Student Lab^®^ system. The protocol consisted of performing 10-s movement periods following a direction indicator; the user followed different positions (up, down, right, and left) and returned to the resting position; this was described as looking straight ahead. A fixation and blink record was also obtained to classify and identify noise signals in the EOG signal acquisition. The resulting file extension was .mat, and this acquisition process is presented in Figure 2 with the signal expressing an amplitude range of −0.3 to 0.3 Volts.

**Figure 3:**
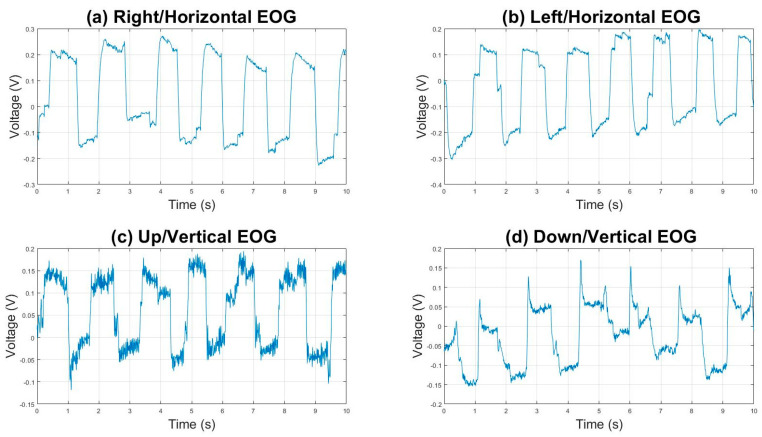
(**a**) Graphic representation of the Electrooculography acquisition of the movement looking to the right, with a threshold ranging from −0.1 to 0.25 volts. (**b**) Signal of eye movement looking to the left, with a threshold of −0.3 to 0.2 volts. Both movements were acquired by the Horizontal EOG channel. (**c**) Eye movement looking up, with a threshold of −0.1 to 0.2 volts. (**d**) Figure representing the downward gaze signal, with a threshold of −0.1 to 0.1 volts. Both movements were acquired by the Vertical EOG channel. All movements acquired within a range of 10 s.

**Figure 4:**
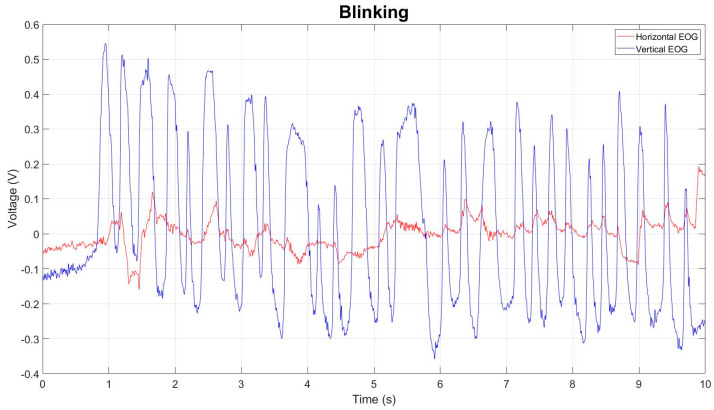
Graphical representation of the Electrooculography acquisition during blinking movement acquisition process; both Vertical and Horizontal EOG channels can be seen in a period of 10 s with a voltage range of −0.4 to 0.5 volts in the vertical EOG channel and a voltage range in the Horizontal channel of −0.1 to 0.2 volts. The Vertical channel shows the greatest amount of change in the signal at the time of flashing.

To validate the performance of each classifier, the EOG signal was divided into Horizontal and Vertical, positive action potentials corresponding to the Right/Horizontal movement (Figure 3a) and Up/Vertical movement (Figure 3b), while negative action potentials corresponded to the Left/Horizontal (Figure 3c) and Down/Vertical movement (Figure 3d); the database contained a fixation and blink record (Figure 4).

**Figure 5:**
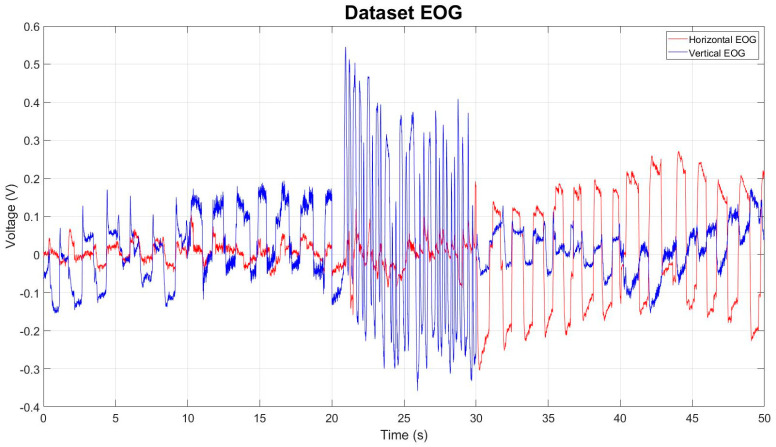
Graphical representation of EOG data set with both Vertical and Horizontal channels, with a total of 32,500 samples for each. Each eye movement (down, up, blink, left and right) is indicated in each of the sample ranges. Each of them was acquired in a 10-s period, keeping the signal in a general range of −0.3 to 0.5 volts.

Figure 5 graphically represents the EOG data set, with a total of 32,500 samples. This is the input used in the processing with the Wavelet Transform.

Table 1 shows the range from the beginning to the end of each eye movement, starting with data 0 to 32.500 of the total samples.

Once a range of eye movements was obtained, the processing was performed with the use of the Wavelet transform, which is explained in Section 2.2, where dimensionality reduction with the transform is explained, as well as its selection with the entropy method. This is followed by Section 2.3, starting with the metrics that were calculated from the general signal for the input to the classifier.

### 2.2. Wavelet

The frequency spectrum of the EOG signal was analyzed, the mathematical tools implemented were Fourier and Wavelet, and the EOG signal had a dynamic behavior, i.e., it varied in time and frequency. By implementing Fourier, the harmonics of the energy spectrum of the signal reduced the information; thus, Wavelet Transform [23,24] was implemented to analyze the different levels of signal frequency obtained in tests of the different movements of eyeballs.

Wavelets are functions used to approximate data with variations, transient and non-stationary phenomena. In the implemented algorithm, data are processed at different resolutions if a signal or function is observed using a wide “data window”. Waveforms are not observed, and such windows are automatically adjusted when changing resolution. Wavelet analysis consists of three steps: decomposition, thresholding and reconstruction.

The continuous wavelet transform (CWT) can be defined as the sum of all scaled and shifted components of the Mother Wavelet’s overall time, as shown in Equation (1).

(1) $$\mathrm{CWT}(a,b)=\frac{1}{\surd a}\int_{-\infty }^{\infty }x\left(t\right)\psi (\frac{t-b}{a})\mathrm{dt},$$

where a is the scale, t is time, b is the displacement and x(t) is the input function and ψ indicates the wavelet function.

The above equation is scaled by ‘a’ and then translated to a second scalar as ‘b’; x represents the EOG signal as a function of time t. Wavelet has a set of families which have members with different parameters for its calculation, and there are two ways to select a Wavelet. The first is to search among the different wavelet families, which have a similar shape to the signal; the second is based on testing with the different wavelets to obtain a smoothing in the signal without losing the points of interest in the original. When a wavelet is selected, it is called the Mother Wavelet, which is represented by Equation (1).

The wavelet detail coefficients indicate the relationship between the signal and the Mother Wavelet, and this ratio allows us to know the frequency components of the signal.

Mother Wavelet was determined using the entropy method; it provides levels [25] that define the amount of disorder of a data set, i.e., the result after its application on the signal, which indicates that the farther it is from the original state, the greater the amount of disorder; therefore, this alters the correlation between the processed signal and the original one.

Thus, this demonstrated that the levels of detail coefficients lost a significant amount of information, indicating that it had a higher level of entropy. The calculation is presented in Equation (2). In [26], methods for obtaining optimization through the Bayesian method of hyperparameters for the classification of stationary signals are presented; however, in this work, it was indicated that with entropy, it was feasible to obtain optimal results of the hyperparameters for classification with supervised algorithms.

(2) $$\mathrm{Entropy}(S)=-\sum \left(p\left(c\right)*\log2\left(p\left(c\right)\right)\right),$$

where S represents the data set in which the entropy is calculated, p(c) is the portion of data points that belong to class c, and to the total number of data points in the set.

**Figure 6:**
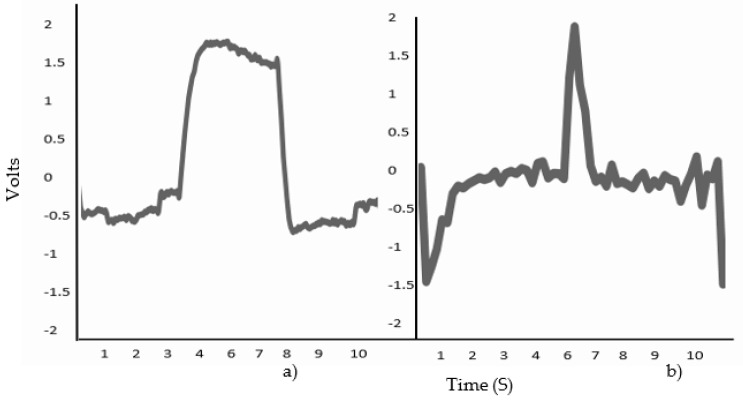
(**a**) The original signal is shown with variations in the signal including its highest point with an amplitude of 2.0 volts; (**b**) The modified signal after going through the calculation of the Bio Reverse Wavelet transform 3.1.

The application of the Mother Wavelet of the EOG signal is shown in Figure 6, where the signal has a different shape from the original; this is given by the detailed coefficients that were applied and allowed important data to be obtained without loss of information.

By obtaining the Wavelet detail level data, we obtain a new signal with the most relevant information of the original one. The results obtained in the frequency spectrum were confirmed in windows of four data; obtaining 508 windows of each EOG channel, the calculation of nine metrics was performed, as shown in Table 2.

Descriptions of each metric that were applied to the signal to obtain data for the classification algorithm.

### 2.3. Classification Algorithms

The implementation of a classification algorithm is one of the requirements to identify offline classes whose membership is known based on training. In this study, offline classification was implemented using three supervised learning algorithms: SVM, KNN and DT.

To compare the best classification results, the classes were labeled (Table 3) to input them into the different algorithms.

Each movement was assigned a class starting from 0 to class 4.

### 2.4. K-Nearest Neighbors Algorithm (KNN)

K-Nearest Neighbor (KNN) is a supervised learning algorithm that uses the proximity of distances for classification by a majority vote, assigning the case to the most common class among its nearest neighbors (K), which is measured by a distance function that then uses the Euclidean equation, as shown in Equation (3).

(3) $$d\left(x,y\right)=\sqrt{\sum_{i=1}^{n}{\left(yi-xi\right)}^{2}}$$

where *x* corresponds to the query point (K) and y to the nearest neighbor to determine which neighbor is the nearest. Application of equation in Algorithm 1 KNN.
**Algorithm 1 KNN****Requiere:** Determine the sample of set data (80% training and 20% testing)Select the value of K**for** each new sample **do**Calculate the distance for simples Determine the set of K neighbors with the closest distance  $d\left(x,y\right)=\sqrt{\overset{n}{\underset{i=1}{\sum }}{\left(yi-xi\right)}^{2}}$The label with the most representative in the set of K neighbors is chosen**end for****Ensure:** Determine the accuracy and the best neighbor

### 2.5. Support Vector Machine Algorithm (SVM)

Support Vector Machines are a supervised learning classifier that works by correlating data in a feature space so that data points can be categorized, even if they are not linearly separated. The features of the new data can be used to define the group to which the new record belongs. To allow some flexibility, the algorithm handles a parameter, C, which controls the trade-off between training errors and rigid margins, thus creating a margin that allows for some errors in the classification, i.e., gives control over the classification errors. The mathematical function used for the transformation is known as a kernel. The polynomial equation shown in Equation (4) was used.

(4) $$K\left(xi,xj\right)={\left(yx_{i}^{T}x_{j}+r\right)}^{d},y>0\;\;\;\;\;\;\;\;\;\;\;\;\;\;\;\;\;$$

where K(xi,xj) corresponds to the matrix of n × n kernel elements, xi,xj corresponds to the feature hyperplane, {(yx}_i^Tx_j+r${)}^{d}$ support vectors act as a separation between classes and represents the data for the measurement and r is the parameter that is being adjusted or calibrated. Application of equation in Algorithm 2 SVM.
**Algorithm 2 SVM****Require**: Determine the sample of set data (80% training and 20% testing)**Ensure**: Determine the accuracySelect kernelSelect the optimal value of the cost and gamma for SVM **while** (topping condition is not met)  $K\left(xi,xj\right)={\left(yx_{i}^{T}x_{j}+r\right)}^{d},y>0\;$**do**Implement SVM train step for each data pointImplement SVM classification for testing data points**end while****accuracy**

### 2.6. Decision Tree Algorithm (DT)

The decision tree algorithm is a machine learning-based algorithm for classification, where an internal node represents a feature, the branch represents a decision rule, and each leaf node represents the result. The top node in a decision tree is known as the root node. It performs a partitioning from the attribute value function and splits the tree in a recursive manner called recursive partitioning. Its structure helps to make decisions from each training set containing labels of each class and predictor variables that can be inspected for a decision or split, which results in a left node and a right node. This starts from the root of the tree and ends at the endpoint in the form of a leaf node giving an output class. Each partition is performed with the clustering of the Gini index, which is presented in Equation (5). Application of equation in Algorithm 3 DT.

(5) $$gdi=1-\sum_{i}p^{2}\left(i\right)$$

where $1-\sum_{i}p^{2}\left(i\right)$ corresponds to the subnode calculation, $p^{2}$ to the sum of the probability squares and i to the data.
**Algorithm 3 DT****Require**: Determine the sample of set data (80% training and 20% testing).Each data are analyzed as the root node of the decision tree is assigned.Each member is assigned a child node. Each of the members of the tree is analyzed and a label is assigned  $gdi=1-\sum_{i}p^{2}\left(i\right)$Predictions are made based on the result of each child with the labeling of each member.**accuracy**

### 2.7. Jaccard Index

The well-known Jaccard similarity algorithm is an algorithm designed to measure similarities between sample sets. There is function-based analysis, which is typically used to study the resemblance of small numbers of sets and, additionally, the analysis of large data sets, calculated by Equation (6).

(6) $$sim_{j}\left(A,B\right)=\frac{\left|A\cap B\right|}{\left|A\cup B\right|}\;$$

where a is the data in group A, b is the data in group B and c is the number of elements present in both groups A and B.

### 2.8. Methodology

The simulations and results were run on a laptop computer with the following computer characteristics: AMD Ryzen 5 3450U with Radeon Vega Mobile Gfx processor with a processor speed of 2.10 to 3.5 GHz and 4 MB processor cache, (2 × 8 dual channel) 16 GB of DDR4 memory at 3000 MHz, a 256 GB Crucial SSD and a video card AMD Radeon 73 graphics card, and the operating system Windows 11 Home Single Language version 22H2 64-bit.

Matlab 2022 was used for signal processing; Python language was used for coding the algorithms as well as the metrics in an online environment. This was implemented on Google Research’s Colaboratory, which allows the execution of different programming languages.

The Train-Test Split method of the Cross-Validation technique was used, which consists of randomly decomposing the data series; this method was very accurate since we evaluated the combinations of training and test data, and the number of iterations depended on the size of the data set; usually, 80% of the data were reserved to be used for training the Machine Learning model. The remaining 20% of the data allowed for testing the algorithm for validation, as applied in this research.

**Figure 7:**
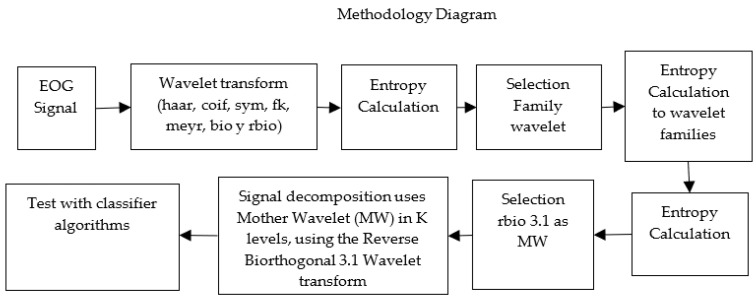
Diagram of the application of the methodology; starting from the original signal, continuing with the wavelet application and the entropy method for the selection of the Mother Wavelet, finishing with the application of the wavelet transform.

A diagram in Figure 7 shows the process followed by the EOG signal with the implemented techniques.

## 3. Results and Discussion

The results obtained from the eye movements in different classification algorithms were analyzed to be defined based on sensitivity and specificity. This, by using the metrics, represented the percentage that corresponded to the most classified values.

We applied the Entropy method to the data obtained from the transform to determine the Wavelet to apply or mother, as shown in the Vertical EOG in Table 4 and Horizontal EOG in Table 5. We analyzed the family wavelet Haar(haar), Coiflets (coif), Symelets (sym), Fejer Korovkin Filters (fk), Discrete Meyer (meyr), Biorthogonal (bio) and Reverse Biorthogonal (rbio), which were the ones that showed similarity with the original signal.

Table 4 shows the results in the rbio family of the Wavelets families.

With the data obtained, it was determined that Wavelet Reverse Biorthogonal would be used in the signal, becoming the Wavelet Mother. Derived from the fact that after the application of entropy, it showed results with lower amounts of information disorder, this family contained members with characteristics that allowed a signal with a level of smoothing to be obtained after its application without losing important data of the original signal. Entropy was applied to each of the 14 members of the Reverse Biorthogonal (rbio) family, showing that member 3.1 was one of those with the least amount of entropy; it this selected as the Wavelet Mother by visual comparison with the other members, showing its five levels of detail coefficient in both EOG channels, which allowed significant data to be obtained from the original signal. Each result is shown in Table 6 for the Vertical EOG channel and in Table 7 for the Horizontal EOG.

Entropy was applied to each of the 14 members of the Reverse Biorthogonal family, and the result with the least amount of entropy was shown in member 3.1. Each result is displayed in Table 6 for the Vertical EOG channel and Table 7 for Horizontal EOG.

The Bior 3.1 family member in the Vertical channel is shown as marked.

Each level of detail coefficient allows the signal to be viewed by segments, finding characteristic points in the parameters. Five levels were analyzed, of which level 4 showed a level of smoothing in the signal, allowing the beginning and end of each of the EOG movements to be found.

### 3.1. Confusion Matrix

**Figure 8:**
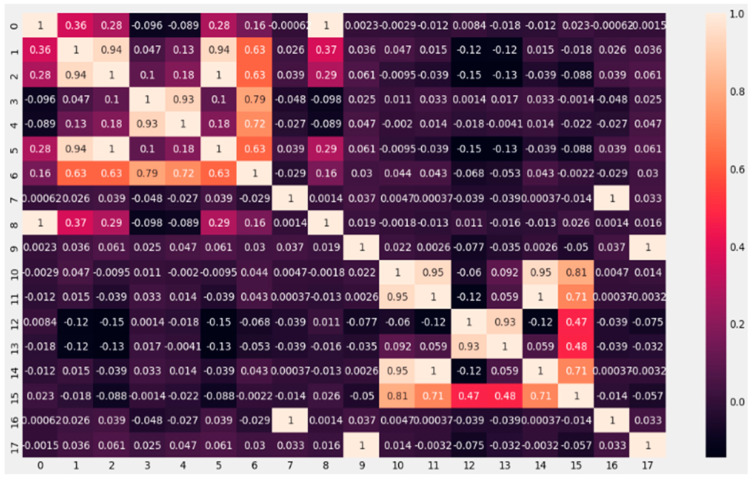
Confusion matrix with 9 features for each Vertical EOG (Table 7) and Horizontal EOG (Table 8), each number from 0 to 17 represents the 18 labels where the name of the feature is indicated: the one that corresponds to each channel and its location number in the matrix.

The confusion matrix was applied to the results of both EOG channels, and the results are shown visually in Figure 8 below.

In the Table 8 shows each of the positive values for the Horizontal EOG channel; negative values, false positives and false negatives for each of the characteristics, these data are important for the calculation of the confusion matrix terms.

Table 9 shows each of the positive values for the Vertical EOG channel, including negative values, false positives, and false negatives for each of the characteristics; these data were important for the calculation of the confusion matrix terms.

VP indicates True Positive, VN indicates True Negative, FN expresses False Negative and FP indicates False Positive.

The results of the aforementioned calculations are shown in Table 10 for the Horizontal EOG channel and Table 11 for the Vertical EOG channel, each corresponding to the result of the characteristic applied to the signal.

The calculation of the data of the confusion matrix in the Horizontal channel is shown.

The results of the calculation for sensitivity, specificity, accuracy, and precision in the Vertical EOG channel are given.

### 3.2. ROC Curve

The results obtained from the ROC curve in the KNN algorithm are presented in Table 12, SVM in Table 13, and DT in Table 14; they show the obtained results of sensitivity and specificity after the input of the complete signal to the classifier to indicate the difference between each term with each result obtained indicating a higher sensitivity index in SVM, checking with the calibration given in this algorithm. 

**Figure 9:**
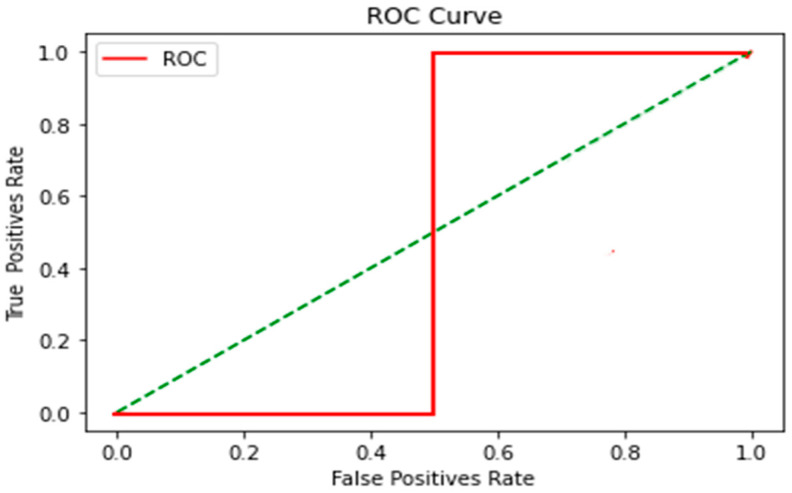
KNN algorithm. Two combinations of neighbors (K) were used, with K = 4 and K = 10, and the nearest neighbor was K = 1. The algorithm training percentage of 80% and 20% testing and with seed = 4. As the signal changed, the ROC curve took a sudden change; this was derived from the fact that the first two movements presented similarities in the voltage amplitude (look up and look down). The eye movement transition was noticed when the blinking movement was performed followed by the left and right movements, derived from the fact that the voltage had a significant range of change at the first movements; this was noticed in the graph when resuming the classification of true positives. For the KNN algorithm it is problematic to have a classification when the signal presents similar data and when it is a large volume. It was also seen that the higher the volume of data, the further away the correct classification due to the number of calculations between distances.

**Figure 10:**
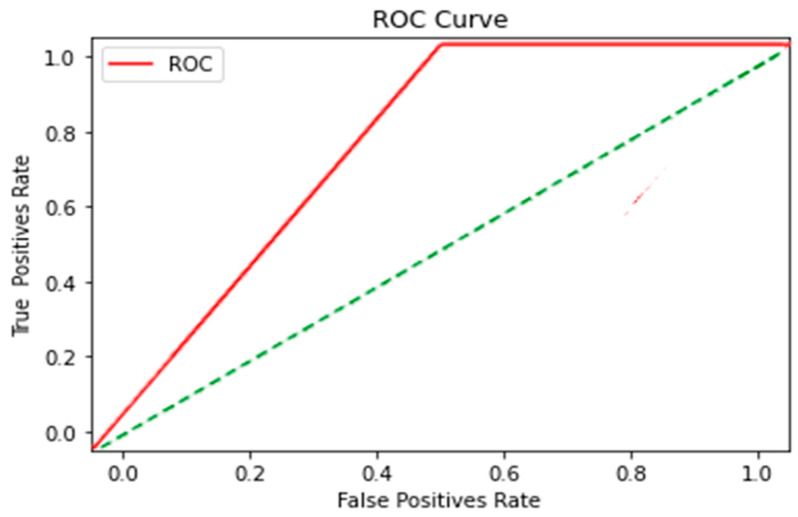
SVM algorithm. A Polynomial Kernel and a regularization parameter C = 14.5 were used, with a training percentage of 80% and 20% of data for testing, with a seed or random state equal to 4.

**Figure 11:**
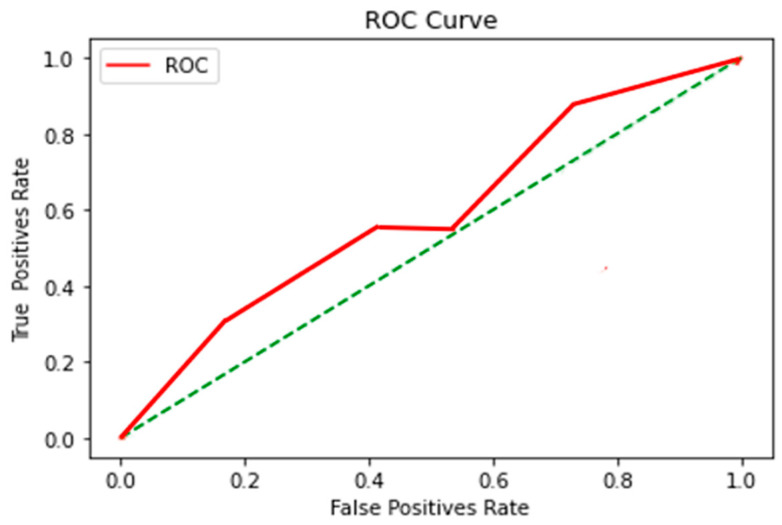
DT algorithm. Implementation of 10 nodes. With a training percentage of 80% and 20% of test data with a seed or random state equal to 4.

These results obtained from the ROC Curve application of KNN, SVM and DT algorithms were obtained and are explained below in Figure 9, Figure 10 and Figure 11 respectively.

(a) KNN

The cutoff point was obtained, and it was visualized as the ROC line changed direction, derived from the variation in the signal data; however, the ROC curve is shown with no success in its classification in the first half of the data, resuming an improvement in the classification in the rest of the signal.

(b) SVM

**Figure 12:**
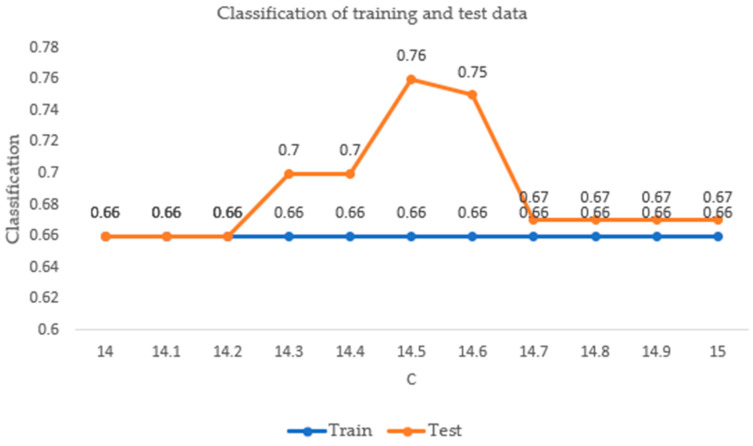
Lustration of training data with test data.

By changing the value of C, the hyperplane of the Kernel was modified. A calibration process was performed (Figure 12), where the value was adjusted, and by choosing a value close to 0, it became closer to some points than others; basically, there was no restriction, and we ended up with a hyperplane that did not classify anything. Since the data were linearly separable, a large C could be used, but this may have been an outlier, and that is why we used a hyperplane very close to the margin with no outlier. We tried several values, and we can say that the selected one provided the freedom to our classifier.

Cross-validation was used with the total data, where 80% was used for training and the remaining 20% for testing; in this graph, from the calibration in C, the qualification improved.

The sensitivity and specificity (Table 14) of the SVM algorithm were calculated with the results obtained with the classification.

The data show a change in the cutoff point; throughout the signal, they were shown above the diagonal that divides the ROC space. The points above the diagonal represent good classification results; they became better as the signal classification progressed.

(c) DT

Table 14 shows the cut-off point sensitivity and specificity of the ROC curve result of the DT algorithm.

Inconsistency was shown in the classification, where no improvement was evident at any point in the signal, indicating that this was an inefficient algorithm for classification.

### 3.3. Jaccard Index

In the Table 15 shows the results of the signal classified with the three classification algorithms with the application of the Jaccard metric, as explained above.

In the analysis of the classification results, SVM obtained the best result compared to the other algorithms in the test column, with 76.9%.

The selection of the KNN, SVM and DT algorithms was derived from issues of explainability since the article focused on the health area.

## 4. Conclusions

Performing routine activities for people with motor disabilities is a problem that impacts their quality of life. For this reason, the research presented in this paper is about the acquisition of two EOG channels that allows data to be acquired from different eye movements, with the help of the implementation of the Wavelet Reverse Biorthogonal 3. 1 to identify the different waveforms of the signal through acquisition windows; this process improved the responses of supervised classifiers KNN, SVM and DT and through the Jaccard index metric the efficiency level of each algorithm was checked. The best result, with a value of 76.9%, was obtained for the SVM classifier in the Jaccard Index metric; according to the state-of-the-art reported, it exceeded the percentage of response in the efficiency of supervised classifiers with values of 69.75% reported in [13]. This translated into better data classification. The program codes and methods implemented in this research are provided at: https://acortar.link/nW8l0s (accessed on 4 May 2023).

For future work, we propose the use of these classifiers by implementing them in different tools, such as a human–machine interface to support assistance and interaction with different users to apply it in the medical area, reducing the response time and the learning curve of inexperienced users.

## Figures and Tables

**Table 1:** Details of reference EOG signal.

Reference EOG Signal Details	Samples Range
Eye movements down direction	0–6500
Eye movements up direction	6001–13,000
Eye blinks	13,001–19,500
Eye movements in the left direction	19,501–26,000
Eye movements in the right direction	26,001–32,500

**Table 2:** Frequency domain EOG signal metrics.

Metric	Definition
Root Mean Square (RMS)	Continuous power without distorting the original signal.
AMP	Amplifies when creating an amplifier object with default property value.
Maximum	Maximum data of the frequency group.
Variance	A measure of dispersion that represents the variability of a data series with respect to its mean.
Covariance	Value reflecting the amount by which any variables vary jointly with respect to their arithmetic means.
Median	Central variable of a data set.
Average	Result obtained by adding several quantities of the amount of data.
Pspectrum	Returns the scale of the frequency spectrum.
Power	Sum of the absolute squares of its time domain samples divided by the length of the signal.

**Table 3:** Labeling of eye movements with their assigned class for input into the classification algorithm.

Movement	Class
Down	0
Blink	1
Up	2
Left	3
Rigth	4

**Table 4:** Results of the Wavelet application on the vertical EOG channel.

Vertical EOG—Entropy							
	Wavelets Families						
Level	haar	coif	sym	fk	meyr	bio	rbio
1	0.6238	0.353	0.6238	0.6894	0.6238	0.6238	0.6238
2	1.4351	0.8195	1.4351	1.2439	1.4351	1.4351	1.4351
3	2.2416	1.9332	2.2416	2.1586	2.2416	2.2416	2.2416
4	2.8955	2.6637	2.8955	2.937	2.8955	2.8955	2.8955
5	3.2258	3.1634	3.2258	3.4161	3.2258	3.2258	3.2258

**Table 5:** Wavelet application results on the Horizontal EOG channel.

Horizontal EOG—Entropy							
	Wavelet Families						
Level	haar	coif	sym	fk	meyr	bio	rbio
1	0.2244	0.9021	0.2244	0.2685	0.2244	0.2244	0.1685
2	0.8362	0.2863	0.8362	0.9736	0.8362	0.8362	0.6736
3	1.5658	1.2524	1.5658	1.4808	1.5658	1.5658	1.4808
4	2.1716	1.9901	2.1716	2.1288	2.1716	2.1716	2.1288
5	2.7221	2.6009	2.7221	2.6985	2.7221	2.7221	2.6985

**Table 6:** Results of the Entropy method on the Reverse Biorthogonal family members of the Vertical EOG channel.

Biorthogonal Wavelet Entropy Vertical														
Reverse Biorthogonal Family Members														
Level	1.3	2.2	2.6	3.1	3.5	3.9	5.5	1.5	2.4	2.8	3.3	3.7	4.4	6.8
1	0.6241	0.0038	0.0038	0.0014	0.0014	0.0014	0.004	0.624	0.0038	0.0038	0.0014	0.0014	0.0033	0.0039
2	1.4513	0.5295	0.5369	0.1826	0.1988	0.2057	0.1663	1.4502	0.5436	0.55	0.1742	0.185	0.2597	0.1443
3	2.3274	1.7774	1.8579	1.6201	1.6899	1.7066	1.6897	2.3452	1.8273	1.8452	1.6731	1.6862	1.7156	1.756
4	2.9393	2.7557	2.7503	2.9252	2.9603	2.9517	2.3844	2.9831	2.7682	2.8042	2.9561	2.9222	2.5359	2.6123
5	3.2659	3.2066	3.1913	3.1462	3.1081	3.1952	2.6238	3.2532	3.1716	3.1734	3.1525	3.1568	2.8598	2.867

**Table 7:** Results of Entropy application to different members of the Reverse Biorthogonal family of the Horizontal EOG channel.

Biorthogonal Wavelet Entropy Horizontal														
Reverse Biorthogonal Family Members														
Level	1.3	2.2	2.6	3.1	3.5	3.9	5.5	1.5	2.4	2.8	3.3	3.7	4.4	6.8
1	0.2244	6.92 ×10	6.92 $\times 10^{-04}$	6.92 $\times 10^{-04}$	6.92 $\times 10^{-04}$	6.92 $\times 10^{-04}$	4.50 $\times 10^{-03}$	2.24 $\times 10^{-01}$	6.9 $\times 10^{-04}$	6.92 $\times 10^{-04}$	6.92 $\times 10^{-04}$	6.92 $\times 10^{-04}$	0.0028	0.0028
2	0.8495	0.1445	0.1493	0.0315	0.0387	0.0434	0.0302	0.8437	0.1402	0.1485	0.0469	0.0535	0.0486	0.0346
3	1.6324	1.1173	1.1747	0.9913	1.0652	1.0674	1.0088	1.6506	1.1558	1.1926	1.043	1.058	1.0587	1.1081
4	2.2723	2.0297	2.101	2.2961	2.2272	2.2652	1.764	2.3231	2.0704	2.1408	2.2949	2.2094	1.8714	1.966
5	2.7988	2.6147	2.6459	2.8491	2.8998	2.8339	2.3469	2.8246	2.6103	2.6527	2.8693	2.8712	2.4207	2.4695

**Table 8:** Parameters and characteristics of the EOG signal Horizontal channel.

Characteristic	Horizontal	TP	TN	FP	FN
Root Mean Square (RMS)	RMSH (0)	1	36.78	0	1.8841
AMP	AMPH (1)	1	83.5	0.36	3.423
Variance	VarianceH (2)	1	76.66	1.22	2.30405
Average	AverageV (3)	1	79.72	0.051	1.966
Medium	MedianaH (4)	1	79.39	1.151	0.8329
Covariance	CovarianzaH (5)	1	16.05	2.5	0.6645
Maximum	MaxH (6)	1	76.30	2.93	0.1949
Pspectrum	PspectrumH (7)	1	80.36	0.0062	1.09084
Power	PowerH (8)	1	82.80	1.937	0.0296

**Table 9:** Parameters and characteristics of the EOG Signal Vertical channel.

Characteristic	Vertical	VP	VN	FP	FN
Root Mean Square (RMS)	RMSV (9)	1	81.87	0.3183	0.9022
AMP	AMPV (10)	1	75.76	0.103	2.7607
Variance	VarianzaV (11)	1	77.28	0.95497	1.65257
Average	PromedioV (12)	1	82.59	0.7812	1.166
Medium	MedianaV (13)	1	79.43	0.0759	0.468
Covariance	CovarianzaV (14)	1	77.77	1.89397	0.71257
Maximum	MaxV(15)	1	73.95	2.9454	0.071
Pspectrum	PspectrumV(16)	1	83.03	0.952127	0.033
Power	PotenciaV(17)	1	81.72	1.1841	0

**Table 10:** Calculation of confusion matrix channel EOG Horizontal EOG.

Characteristic	Horizontal	Sensitivity	Specificity	Accuracy	Precision
Root Mean Square (RMS)	RMSH (0)	34.72	100	93.73	100
AMP	AMPH (1)	22.60	99.57	95.42	73.52
Variance	VarianzaH (2)	30.26	98.43	95.43	45.04
Average	PromedioV (3)	33.78	99.93	97.35	95.14
Medium	MedianaH (4)	54.64	98.57	97.38	39.84
Covariance	CovarianzaH (5)	60.24	86.52	80.41	28.57
Maximum	MaxH (6)	83.68	96.30	95.87	25.44
Pspectrum	PspectrumH (7)	47.84	99.99	98.45	34.12
Power	PotenciaH (8)	98.03	97.72	97.55	34.12

**Table 11:** Calculation of confusion matrix channel EOG Vertical EOG.

Characteristic	Vertical	Sensitivity	Specificity	Accuracy	Precision
Root Mean Square (RMS)	RMSV (9)	52.63	99.62	98.37	45.87
AMP	AMPV (10)	26.59	99.86	96.14	90.66
Variance	VarianzaV (11)	37.73	98.78	96.54	51.28
Average	PromedioV (12)	46.16	99.06	97.55	56.14
Medium	MedianaV (13)	68.11	99.90	97.26	34.55
Covariance	CovarianzaV (14)	58.47	97.62	96.57	34.60
Maximum	MaxV (15)	93.37	96.16	95.87	25.34
Pspectrum	PspectrumV (16)	96.8054	98.86	98.67	51.28
Power	PotenciaV (17)	45.87	98.57	98.39	45.87

**Table 12:** Sensitivity and specificity of algorithm KNN.

Cutting Point	Sensitivity	Specificity
0.5	50%	50%

**Table 13:** Sensitivity and specificity of SVM algorithm of the best classification.

Cutting Point	Sensitivity	Specificity
0.5	50%	50%

**Table 14:** Sensitivity and specificity of algorithm DT.

Cutting Point	Sensitivity	Specificity
0.43	57%	43%

**Table 15:** Comparative results of the DT Algorithm, SVM and KNN in the Jaccard Index.

Algorithm	Train	Test
DT	0.5	0.60526
SVM	0.66666	0.76949
KNN	0.41176	0.69458

## Data Availability

Information on this research can be obtained from the following link https://acortar.link/nW8l0s (accessed on 4 May 2023).
